# African Swine Fever Virus I267L Is a Hemorrhage-Related Gene Based on Transcriptome Analysis

**DOI:** 10.3390/microorganisms12020400

**Published:** 2024-02-17

**Authors:** Yuan Wen, Xianghan Duan, Jingjing Ren, Jing Zhang, Guiquan Guan, Yi Ru, Dan Li, Haixue Zheng

**Affiliations:** 1 State Key Laboratory for Animal Disease Control and Prevention, College of Veterinary Medicine, Lanzhou University, Lanzhou Veterinary Research Institute, Chinese Academy of Agricultural Sciences, Lanzhou 730000, China; yuanwenbiology@outlook.com (Y.W.); duanxh1999@126.com (X.D.); yashangzhuo@163.com (J.R.); 13389082893@163.com (J.Z.); guanguiquan@caas.cn (G.G.); ruyi@caas.cn (Y.R.); 2 Gansu Province Research Center for Basic Disciplines of Pathogen Biology, Lanzhou 730000, China

**Keywords:** African swine fever virus, I267L, hemorrhage, F3, tissue factor

## Abstract

African swine fever (ASF) is an acute and severe disease transmitted among domestic pigs and wild boars. This disease is notorious for its high mortality rate and has caused great losses to the world’s pig industry in the past few years. After infection, pigs can develop symptoms such as high fever, inflammation, and acute hemorrhage, finally leading to death. African swine fever virus (ASFV) is the causal agent of ASF; it is a large DNA virus with 150–200 genes. Elucidating the functions of each gene could provide insightful information for developing prevention and control methods. Herein, to investigate the function of I267L, porcine alveolar macrophages (PAMs) infected with an I267L-deleted ASFV strain (named ∆I267L) and wild-type ASFV for 18 h and 36 h were taken for transcriptome sequencing (RNA-seq). The most distinct different gene that appeared at both 18 hpi (hours post-infection) and 36 hpi was F3; it is the key link between inflammation and coagulation cascades. KEGG analysis (Kyoto encyclopedia of genes and genomes analysis) revealed the complement and coagulation cascades were also significantly affected at 18 hpi. Genes associated with the immune response were also highly enriched with the deletion of I267L. RNA-seq results were validated through RT-qPCR. Further experiments confirmed that ASFV infection could suppress the induction of F3 through TNF-α, while I267L deletion partially impaired this suppression. These results suggest that I267L is a pathogenicity-associated gene that modulates the hemorrhages of ASF by suppressing F3 expression. This study provides new insights into the molecular mechanisms of ASFV pathogenicity and potential targets for ASFV prevention and control.

## 1. Introduction

African swine fever (ASF) is notorious for its acute infection with a high mortality rate, thereby posing a great threat to the global pig industry. It was first reported in Kenya in 1921 [1] and was naturally transmitted among warthogs and bush pigs in sub-Saharan Africa, with soft ticks being its intermediate host [2]. Though it is mild in wild boars in this area, domestic pigs [3] and wild boars in other areas can have symptoms of a high fever and acute hemorrhages; they die in several days, with a mortality rate of up to 100% [4,5]. Up to now, no commercial vaccine has been approved, except in Vietnam [6].

ASF has spread out of Africa twice. The first instance occurred in Europe in 1957 and then in the Caribbean and Brazil in the 1960s [7]. This outbreak was cleared (except in Sardinia) in the 1990s through large scale eradication. In 2007, the disease spread to Georgia and then to nearby countries such as Russia, Ukraine, Poland, Belgium, and Germany [8]. In 2018, the situation worsened when ASF broke out in China, as China had half of the world’s pig population at that time [9]. Subsequently, based on World Organization for Animal Health (OIE) data, ASF was continually transmitted to other areas including East Asia, South Asia, Southeast Asia, Oceania, and Latin America (https://www.woah.org/en/disease/african-swine-fever/#ui-id-2, accessed on 23 December 2023).

African swine fever virus (ASFV) is the causal agent of ASF; it is a large DNA virus that replicates in the cytoplasm of infected cells and is the only member of the Asfarviridae family [10]. Naturally, it mainly replicates in mononuclear phagocytic cells [11], but it can also use endothelial cells [12] and neutrophils as its permissive cells [13]. The genome of this virus contains more than 170 kilo-nucleotides and encodes 150–200 open reading frames (ORFs) [14,15]. The genes at both ends of its genome are varied and thus can change the genome length. Nowadays, with ASFV spreading to more and more countries, more and more research is focusing on the virus.

A number of ORFs have been studied, and a series of genes with different functions have been identified such as E183L, B646L, XP124L, CP2475L, E120R, which are essential for virus morphogenesis [16]. A238L can prevent the activation of nuclear factor-kappa B (NF-κB)-dependent gene transcription; DP96R can inhibit cGAS–STING pathways to block the production of interferon (IFN); A179L is a putative BCL-2-like protein and thus can bind to all core death-inducing mammalian Bcl-2 proteins to inhibit apoptosis and autophagy [16]. Given that many genes in its genome remain unexplored, extensive research of ASFV is still needed. Some genes, despite being studied before, could have multiple functions. For instance, MGF505-7R can simultaneously inhibit the cGAS–STING pathway, IFN-γ-mediated JAK-STAT1 signaling pathway, and IL-1β maturation [17,18,19]. E248R was first identified as a structural protein vital for virus invasion but was later found to inhibit IFN production [20,21]. Additionally, the mechanisms of many ASF symptoms are still unknown. For example, some pigs can develop a chronic infection [22] that allows the virus to remain alive in a pig farm for a long time.

ASFV can cause hemorrhages in the skin, joints, and different internal organs, thus leading to death. This symptom of hemorrhages is always caused by the excessive production of inflammatory factors, which is named a cytokine storm. Former studies have indicated that after ASFV infection, pigs may develop a cytokine storm [3,23]. The reason why a cytokine storm causes hemorrhages has been explored before. The most important factor linking inflammation and coagulation cascades was a protein called tissue factor (TF) [24] that is encoded by the F3 gene and serves as the initial factor of extrinsic coagulation cascades. TF is generally expressed on cells around blood vessels, such as adventitial fibroblasts and epithelial cells, and has no contact with blood [25]. After the vessel is injured, tissue factor outside the vessels can sense the coagulation factor VII of the blood as well as initial extrinsic coagulation pathways. TF is not constitutively expressed on monocyte–macrophage cells (except alveolar macrophages) and endothelium cells [26]. Inflammation cytokines, such as tumor necrosis factor (TNF)-α and interleukin (IL)-6, could induce the production of TF in monocyte–macrophage cells and endothelium cells to initiate coagulation cascades. As the cytokines always act on the nearby cells (in some instances, they can also act on distant cells), this action is always limited to a local area. The collaboration between inflammation and clotting is a basic survival strategy in vertebrates for walling off the damaged and infected tissues from the rest of the host [27]. However, for systemic inflammation, such as that from an ASFV infection, inflammation cytokines are overwhelmingly produced and excessively initiate coagulation cascades through the symptom of disseminated intravascular coagulation (DIC) [28] and vascular thrombosis [29]. This process can exhaust coagulation factors and lead the host to die from hemorrhages. Apart from the initiation of coagulation cascades, TF can also regulate the immune system. In fact, TF is a member of the human class II cytokine receptor family and displays high homology with the IFN-γ receptor [30]. The FVIIa:TF complex induces proinflammatory effects in macrophages [31], the driven gene transcription of inflammatory factors, such as IL-8, IL-1, and the leukemia inhibitory factor (LIF) observed in keratinocytes [32].

It benefits ASFV to inhibit inflammation and coagulation cascades to allow the host to survive, thereby producing and shedding more viruses. Previous studies identified a number of ASFV genes, such as I329L, DP96R, L83L, F317L, A238L, and so on, that could inhibit the identified inflammation [33]; however, no gene in ASFV has been reported to able to affect coagulation cascades directly.

The I267L gene encodes 267 amino acids and is located at the right end of the genome. Previous research on this gene has yielded varying conclusions. One study using the ASFV SY18 strain found that I267L had no impact on viral replication and virulence. The deletion of this gene did not change the replication and pathology of the virus [34]. Another study using ASFV CN/GS/2018 identified that I267L was an important virulent factor due to its inhibition of RNA polymerase III-RIG-I-mediated IFN-β production [35]. However, the ASFV SY18 strain and ASFV CN/GS/2018 are closely related and share the same I267L sequence. Therefore, whether I267L was a pathology-related gene needs further investigation.

As RNA-Seq analysis is a useful tool for studying the function of the virus gene, we have used this method to analyze the transcriptome difference of the ASFV- and an I267L-deleted ASFV strain (named ∆I267L)- infected porcine alveolar macrophages (PAMs) so as to support more data about I267L’s function. The strains in our studies are the same as one previous study [35]. Our results reveal that ASFV and ∆I267L have similar replication kinetics; RNA-Seq results provide information indicating that I267L could influence the immune system and coagulation cascades. The most distinct gene affected by the deletion of I267L was coagulation factor III (F3, also known as tissue factor, TF), which is the most important link between inflammation and hemorrhages [36]. Compared with ∆I267L-infected cells, ASFV can significantly inhibit TNF-α-induced F3 expression. Additionally, our data also show that I267L could affect the immune system. These findings suggest that I267L is a virulence-associated gene that could affect hemorrhage and immune response in ASFV-infected pigs and provide more information on the pathogenicity of ASFV.

## 2. Materials and Methods

### 2.1. Cells and the Virus

PAMs were isolated from three Duroc-landrace-yorkshire swine through bronchoalveolar lavage, as previously described [37]. PAMs were cultured in RPMI 1640 (Gibco, manufactured at ThermoFisher Biochemical Products(Beijing) Co., Ltd.; Beijing, China) and supplemented with 10% fetal bovine serum (Gibco) and 1% penicillin-streptomycin (ThermoFisher Biochemical Products(Beijing) Co., Ltd.; Beijing, China) at 37 °C with 5% CO_2_.

ASFV isolate CN/GS/2018 that propagates on PAMs was used as ASFV, and ASFV–∆I267L was an I267L-deleted strain of CN/GS/2018, as previously studied [35]; ASFV–GFP was constructed using a GFP gene (also with a p72 promotor) inserted between MGF-110-5L-6L and MGF-110-1R, which have a similar replication kinetic as that of ASFV. Titration of the virus was performed using a hemadsorption assay; the results were presented as Fifty percent hemadsorption doses (HAD_50_) per milliliter.

All animals were handled in strict accordance with good animal practice according to the animal ethics procedures and guidelines of the People’s Republic of China, and the study was approved by the animal ethics committee of Lanzhou Veterinary Research Institute, Chinese Academy of Agricultural Sciences (approval numbers: No. LVRIAEC-2022-049, approved on 7 October 2022; No. LVRIAEC-2023-067, approved on 22 October 2023; No. LVRIAEC-2023-075, approved on 24 November 2023; and No. LVRIAEC-2022-049, approved on 22 December 2023).

### 2.2. RNA-Seq and Data Analysis

PAMs isolated from the three pigs were seeded in 10 cm diameter dishes and infected with ASFV or with ASFV–∆I267L at a multiplicity of infection (MOI) of five, while uninfected PAMs were set as controls. The viruses containing the medium were replaced with a normal medium at 1 h post-infection (hpi) to ensure that the infections were synchronized. Cells were collected at 18h and 36 h post-infection. PAMs from different pigs with the same virus and the same infection times were pooled as one sample for further RNA-seq analysis.

RNA was extracted using TRIZOL reagent (ThermoFisher Biochemical Products, Beijing, China) according to the manufacturer’s protocol. The yield of RNA was determined using a NanoDrop 2000 spectrophotometer (Thermo Fisher Scientific, Madison, WI, USA), and the integrity was evaluated using agarose gel electrophoresis stained with ethidium bromide. The mRNA was then isolated from the total RNA using VAHTS mRNA capture beads (N401-02, Vazyme Biotech, Nanjing, China) according to the manufacturer’s protocol.

The mRNA underwent reverse transcription to cDNA using a VAHTS Stranded mRNA-seq Library Prep Kit for Illumina V2 (NR612-02, Vazyme Biotech, Nanjing, China) with 1 μg of mRNA; it was then amplified using the VAHTS HiFi Amplification Mix (NR616-01, Vazyme Biotech, Nanjing, China) and purified with VAHTS DNA Clean Beads (N411-03-AA, Vazyme Biotech, Nanjing, China).

After construction, cDNA libraries were sequenced on a Illumina Novaseq 6000 platform, and 150 bp paired-end reads were generated. Raw reads of a fastq format were firstly processed using fastp to remove adaptor sequences and discard reads of <50 nt after trimming to obtain clean reads.

Hisat2 (version 2.1.0) was used to align the clean reads with a swine genome database and a swine mRNA database to obtain swine genome location information and transcript information. Clean reads were also aligned with the ASFV genome through Histat2 to obtain ASFV genome information.

The files from Hisat2 were subsequently aligned to a swine gene annotation file and the ASFV genome database using HTSeq-count to obtain the read counts of each gene; the gene expression levels were normalized through the FPKM (Fragments Per Kilobase of exon model per Million mapped fragments; FPKM = total exon Fragments/[exon length(KB) × Millions].

Principal Component Analysis (PCA) analysis was performed using R (v 3.2.0) to evaluate the biological duplication of sample.

Differentially expressed genes (DEGs) analysis was performed through DEGSeq [38], which could analyze the data with no biological replication. Genes with |log_2_(Fold-change)| > 1 and a Benjamini–Hochberg-adjusted *p*-value (q-value) < 0.05 [39] were considered to be DEGs.

A ClusterProfiler program was used for GO and KEGG enrichment analysis using GO database and KEGG databases; the species was *Sus scrofa*. The method of gene ontology (GO) and Kyoto encyclopedia of genes and genomes (KEGG) enrichment analysis was conducted by counting the number of DEGs included in each GO and KEGG term using Fisher’s precision probability test to calculate the *p* value of each term. The GO and KEGG terms with a *p* value of <0.05 was defined as significantly enriched.

The key software and parameters in the RNA-seq analysis are shown in Table 1.

### 2.3. Reference Database

The referenced swine genome database was NCBI_Sscrofa11.1 and is available at ftp://ftp.ncbi.nlm.nih.gov/genomes/all/GCF/000/003/025/GCF_000003025.6_Sscrofa11.1/GCF_000003025.6_Sscrofa11.1_genomic.fna.gz, accessed on 7 November 2022.

The referenced swine mRNA database was NCBI_Sscrofa11.1 and is available at ftp://ftp.ncbi.nlm.nih.gov/gnomes/all/GCF/000/003/025/GCF_000003025.6_Sscrofa11.1/GCF_000003025.6_SscPAMrofa11.1_rna.fna.gz, accessed on 7 November 2022.

The referenced swine gene annotation file was NCBI_Sscrofa11.1 and is available at ftp://ftp.ncbi.nlm.nih.gov/genomes/all/GCF/000/003/025/GCF_000003025.6_Sscrofa11.1/GCF_000003025.6_Sscrofa11.1_genomic.gff.gz, accessed on 7 November 2022.

The referenced African swine fever virus genome database was from ASFV Georgia 2007/1, GenBank accession number: FR682468.2, accessed on 7 March 2023.

The GO database is available at http://geneontology.org/, accessed on 7 November 2022.

The KEGG database is available at http://www.genome.jp/kegg/, accessed on 7 November 2022.

### 2.4. RNA Isolation and Quantitative Real-Time Reverse Transcription PCR(RT-qPCR)

RNA was extracted using TRIzol reagent (ThermoFisher Biochemical Products, Beijing, China). The cDNA was synthesized using a Prime Script RT reagent kit (TaKaRa Bio Inc, Dalian, China) and then used as the template for real-time qPCR using the TB Green Premix Ex Taq mix kit (TaKaRa Bio Inc., Dalian, China). RT-qPCR assays were performed on a C1000 Touch Thermal Cycler (Bio-Rad Laboratories, Singapore) and the software used was Bio-Rad CFX Manager 3.1(Bio-Rad Laboratories, Singapore) for 40 cycles of denaturation (96 °C for 5 s), reannealing, and extension (60 °C for 30 s). The relative mRNA level of these genes was normalized to the porcine GAPDH mRNA level. mRNA was calculated and normalized based on the comparative cycle threshold (2^−^^ΔΔCt^) method [40]. The primers of each gene are shown in Table 2.

### 2.5. Virus Titration

ASFV CN/GS/2018 and the ΔI267L strain were quantified by using hemadsorption assays, as described previously [41], with minor modifications. PAMs were seeded in 96-well plates. Viruses were diluted through twofold serial dilution and added to each well; each dilution sample was measured in eight wells. Then, anti-coagulated whole blood cells of healthy pigs were washed with sterilized PBS (pH 7.2), centrifuged at 350× *g* 2 times, and re-suspended in PBS with 100 times the precipitation volume afterward. A total of 100 μL of re-suspended solution was added to each well. Adsorption of red blood cells in each well was observed at 7 days. The wells containing hemadsorption phenomenon were recorded as positive, or they would otherwise be recorded as negative. Fifty percent hemadsorption doses (HAD_50_) were calculated according to the Reed–Muench method [42].

### 2.6. Flow Cytometry

The cells were infected with ∆I267L or ASFV–GFP. After harvesting, 10^6^ PAMs were centrifuged and suspended in 800 μL of PBS with 1% bovine serum albumin; the LSRFortessa (BD bioscience, San Jose, CA, USA) flow cytometer was used to calculate the PAMs with GFP fluorescent. A total of 50,000 cells were counted for each of the samples. The fluorescent numbers at 72 hpi were deemed to be 100 percent, and the GFP cells in other samples were normalized with the same sample at 72 hpi.

### 2.7. TNF-α Stimulation

PAMs were seeded in 6-well plates for 12 h and then infected with ASFV CN/GS/2018 or the ΔI267L strain at a MOI of five. TNF-α (recombinant human TNF-alpha, Peprotech, Rocky Hill, CT, USA) was added at 6 hpi at a concentration of 10 pg/μL. PAMs were harvested at 18 hpi. Uninfected cells stimulated with or without TNF-α were set as controls.

### 2.8. Biosafety Statement and Facility

All ASF live virus experiments were carried out in the biosafety level 3 (P3) facility of Lanzhou Veterinary Research Institute of the Chinese Academy of Agricultural Sciences and approved by the Ministry of Agriculture and Rural Affairs and the China National Accreditation Service for Conformity Assessment.

### 2.9. Data Availability

The raw sequencing data are available on the sequence read archive (SRA) database under BioProject number PRJNA980651.

## 3. Results

### 3.1. Growth Kinetic of the *ΔI267L Strain*

**Figure 1:**
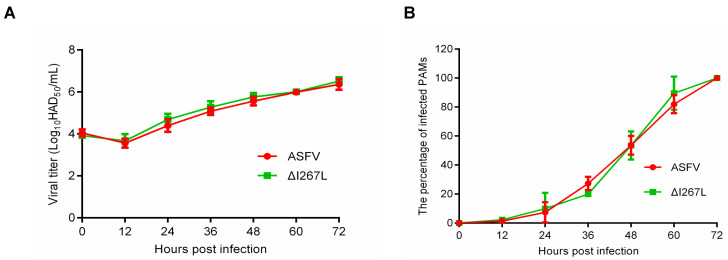
(**A**) Kinetic replication curves of African swine fever virus (ASFV) and I267L-deleted ASFV strain (∆I267L). Three lots of porcine alveolar macrophages (PAMs) were seeded in a 6-well platelet and infected, respectively, with a virus at a multiplicity of infection (MOI) of 0.01 and washed 2 h later. Then, 100 μL supernatants were collected at 0, 12, 24, 36, 48, 60, and 72 hpi to measure the virus titers through a Fifty percent hemadsorption doses (HAD_50_) assay. (**B**) The percentage of infected PAMs. Three lots of PAMs were seeded in a 6-well platelet; the wells were, respectively, infected with a virus at a MOI of 0.01 at 0, 12, 24, 36, 48, 60; cells were all harvested at 72 h.

To determine the influence of I267L deletion on ASFV replication, we measured the growth kinetic of the ΔI267L strain and that of ASFV. Results showed that ΔI267L and ASFV displayed similar growth kinetics, suggesting that I267L gene deletion does not affect ASFV replication (Figure 1A). Flow cytometry results showed that the ΔI267L strain- and ASFV-infected cell numbers grew at a similar speed (Figure 1B).

### 3.2. RNA-Seq and Sequencing Data Quality Analysis

To observe the durable effects of the deletion of I267L, the cell harvesting time ought to cover the whole lifespan of the infected PAMs. Former studies proved that I267L continually transcripts at 6 h post-infection [34,35]. So as to give the gene enough time to influence the transcriptome of cells, we set 18 h post-infection as the first time point to harvest cells.

According to previous studies, after infection with ASFV at a MOI of five, the cell viability rate was about 70% at 24 hpi and 50% at 48 hpi [43]. In our lab, we also found that the infected PAMs could survive for more than 48 h, so we harvested the PAMs at 36 hpi to analyze durable transcription changes in the infected cells.

Each sample was subjected to RNA-seq analysis. The statistics of the sequencing data of each sample are shown in Table 3. Results showed that after the removal of low-quality reads, over 47 M clean reads were generated for each sample. The mapped reads ranged from 76.7% to 95.15%, with an average rate of 89.23%. Additionally, around 14,000 genes were detected in each sample.

**Figure 2:**
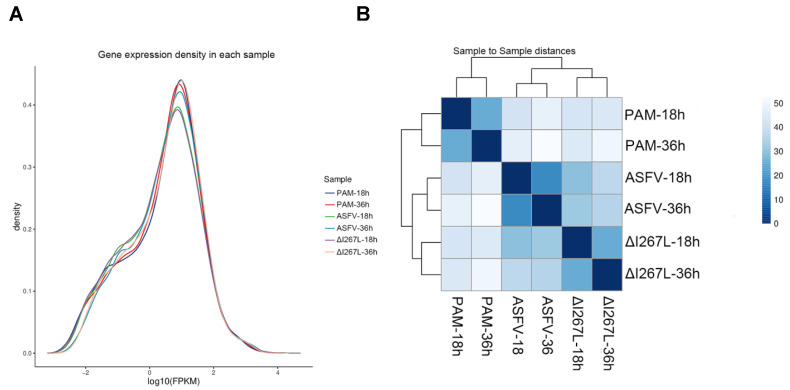
Sample data pattern analysis. (**A**) Gene expression density in each sample. The area under the curve is one. (**B**) Sample-to-sample distances. All the genes identified in the sample and their numbers are listed to test their correlation. The deep color means high correlation with little distance, while the light color means low correlation with far distance.

The results showed that gene expression distribution was similar among all six samples (Figure 2A). The correlation of the samples is shown in Figure 2B. We observed that infection time influences the transcriptome pattern more than the deletion of I267L does.

### 3.3. Differentially Expressed Genes

**Figure 3:**
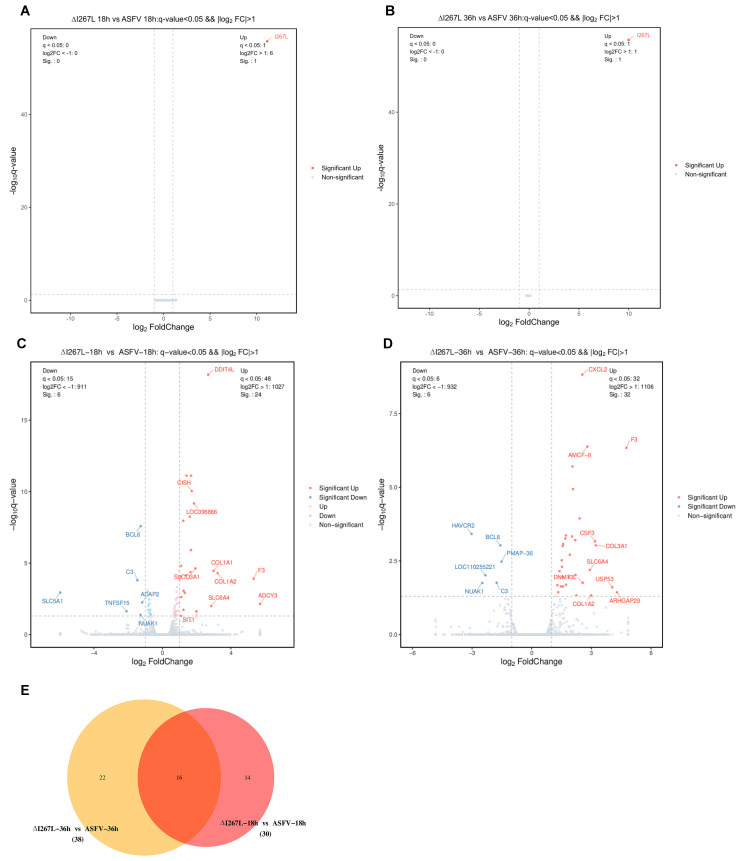
Differentially expressed genes (DEGs) analysis. (**A**) Volcano plot of ΔI267L-infected vs. ASFV-infected PAMs at 18 hpi (ASFV genes); (**B**) volcano plot of ΔI267L-infected vs. ASFV-infected PAMs at 36 hpi plot (ASFV genes); (**C**) volcano plot of ΔI267L-infected vs. ASFV-infected PAMs at 18 hpi (host genes); (**D**) volcano plot of ΔI267L-infected vs. ASFV-infected PAMs at 36 hpi (host genes); (**E**) Venn diagram of the ASFV-infected group, ΔI267L-infected group, and uninfected group (host genes)(Red area: DEGs appeared at ΔI267L infected PAMs vs ASFV infected PAMs at both 18 h and 36 h; pink area: DEGs only appeared at ΔI267L infected PAMs vs ASFV infected PAMs at 18 h; yellow area: DEGs only appeared at ΔI267L infected PAMs vs ASFV infected PAMs at 36 h).

The clean reads were firstly aligned with the ASFV genome; no DEGs, other than I267L, were identified at both 18 hpi and 36 hpi between ASFV-infected PAMs and ΔI267L-infected PAMs (Figure 3A,B).

Compared with ASFV-infected PAMs, ΔI267L-infected PAMs had 30 DEGs at 18 hpi (Figure 3C). ADCY3 had the largest foldchange value among the up-regulated DEGs, while SLC5A1 was the most distinct down-regulated DEG. The second up-regulated DEG was F3, which is the initial factor of extrinsic coagulation cascades and also serves as the most important link between cytokines and coagulation cascades [24]. The third and fourth up-regulated genes were COL1A2 (collagen type I alpha 2 chain) and COL1A1 (collagen type I alpha 1 chain). These two genes are components of collagen, which are also related to platelet activation [44]. A total of 38 DEGs were identified at 36 hpi, with the most up-regulated DEG being F3 (Figure 3D). A total of 16 DEGs were identified at both time points (Figure 3E). The foldchange values of these 16 DEGs are shown in Supplementary Table S1, with F3 having the largest foldchange values at both time points.

### 3.4. Gene Ontology Analysis of DEGs

**Figure 4:**
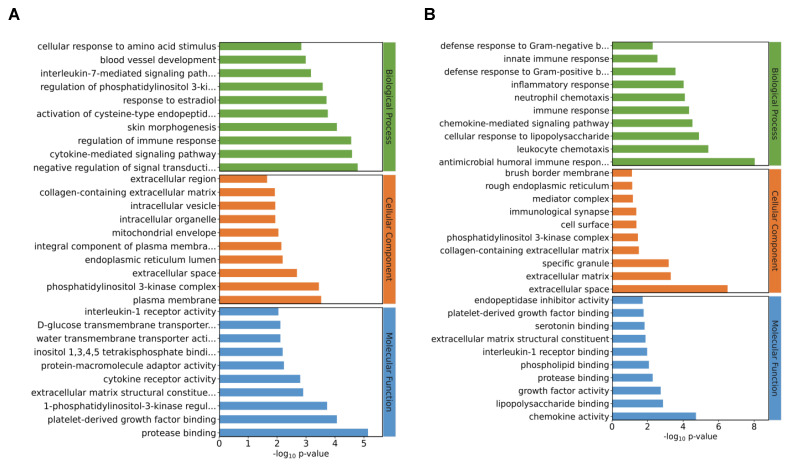
Gene Ontology (GO) analysis of the DEGs. (**A**) Each of the top 10 terms used for GO analysis of the ΔI267L-infected group vs. the ASFV-infected PAM at 18 hpi. (**B**) Each of the top 10 terms used for GO analysis of the ΔI267L-infected group vs. the ASFV-infected PAM at 36 hpi. The terms included are as follows: interleukin-7-mediated signaling path… (interleukin-7-mediated signaling pathway); regulation of phosphatidylinositol 3-ki… (regulation of phosphatidylinositol 3-kinase activity); activation of cysteine-type endopeptid… (activation of cysteine-type endopeptidase activity involved in apoptotic process); negative regulation of signal transducti… (negative regulation of signal transduction); integral component of plasma membra… (integral component of plasma membrane); D-glucose transmembrane transporter… (D-glucose transmembrane transporter activity); inositol 1,3,4,5 tetrakisphosphate bindi… (inositol 1,3,4,5 tetrakisphosphate binding); extracellular matrix structural constitue (extracellular matrix structural constituent conferring tensile strength); 1-phosphatidylinositol-3-kinase regul… (1-phosphatidylinositol-3-kinase regulator activity); defense response to Gram-negative b… (defense response to Gram-negative bacterium); defense response to Gram-positive b… (defense response to Gram-positive bacterium); antimicrobial humoral immune respon… (antimicrobial humoral immune response mediated by antimicrobial peptide).

After the identification of the DEGs, GO analysis was performed. Results showed that the transcriptome changes through the deletion of I267l were mainly related to biological processes (BPs) as well as cellular components at both 18 hpi (Supplementary Figure S1A) and 36 hpi (Supplementary Figure S1B). At 18 hpi, among the top 10 detailed terms of biological processes, 3 were in relation to the immune system, including the cytokine-mediated signaling pathway, regulation of immune response, and interleukin-7-mediated signaling pathway (Figure 4A). At 36 hpi, all the top 10 detailed terms of BPs were in relation to either immune reaction or infection (Figure 4B). These analyses indicated that I267L had the function of modulating the immune reaction of the host and thus may be a pathogenic gene that is worthy of further study.

### 3.5. Kyoto Encyclopedia of Genes and Genomes Analysis of DEGs

**Figure 5:**
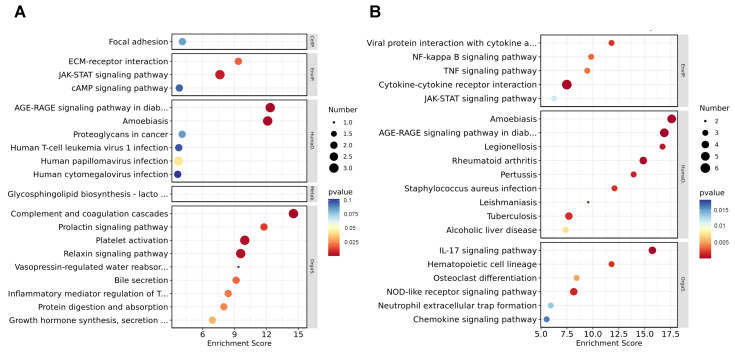
Kyoto Encyclopedia of Genes and Genomes (KEGG) analysis of the DEGs. (**A**) Top 20 up-regulated pathways for the ASFV-infected group vs. uninfected group. The pathways were listed according to the *p* values without being filtered through *p* < 0.05. (**B**) Top 20 down-regulated pathways for the ASFV-infected group vs. the uninfected group. The pathways were listed according to the *p* values without being filtered through *p* < 0.05. The pathways included are described as follows: AGE–RAGE signaling pathway in diab… (AGE–RAGE signaling pathway in diabetic complications); Glycosphingolipid biosynthesis—lacto… (Glycosphingolipid biosynthesis—lacto- and neolacto-series); Vasopressin-regulated water reabsorb… (Vasopressin-regulated water reabsorption); Inflammatory mediator regulation of T… (Inflammatory mediator regulation of TRP channels); Growth hormone synthesis, secretion… (Growth hormone synthesis, secretion, and action); Viral protein interaction with cytokine a… (Viral protein interaction with cytokine and cytokine receptor).

To further study the biological process influenced by I267L, we used KEGG analysis to analyze pathway enrichment. We found that the DEGs between I267L- and ASFV-infected PAMs were mainly linked to signal transduction, the immune system, and infection-related diseases at both 18 hpi (Supplementary Figure S2A) and 36 hpi (Supplementary Figure S2B). At 18 hpi, the most observed enrichment pathway comprised complement and coagulation cascades (Figure 5A), which may be related with the hemorrhages of ASFV-infected pigs. The second enrichment pathway was the AGE–RAGE signaling pathway in diabetes complications, which also correlated with TNF-α signaling and inflammation [45,46]. Other pathways regarding immune response, such as the JAK-STAT signaling pathway, were significantly affected. At 36 hpi, the amoebiasis and AGE–RAGE signaling pathway were also highly enriched; other immune response-related pathways, such as the JAK-STAT pathway, NF-κB signaling pathway, and the IL-17 signaling pathway were also significantly affected (Figure 5B). Combined with the GO analysis, it seems that I267L could influence the immune system more as an infection progresses.

### 3.6. Validation of RNA-Seq Results

**Figure 6:**
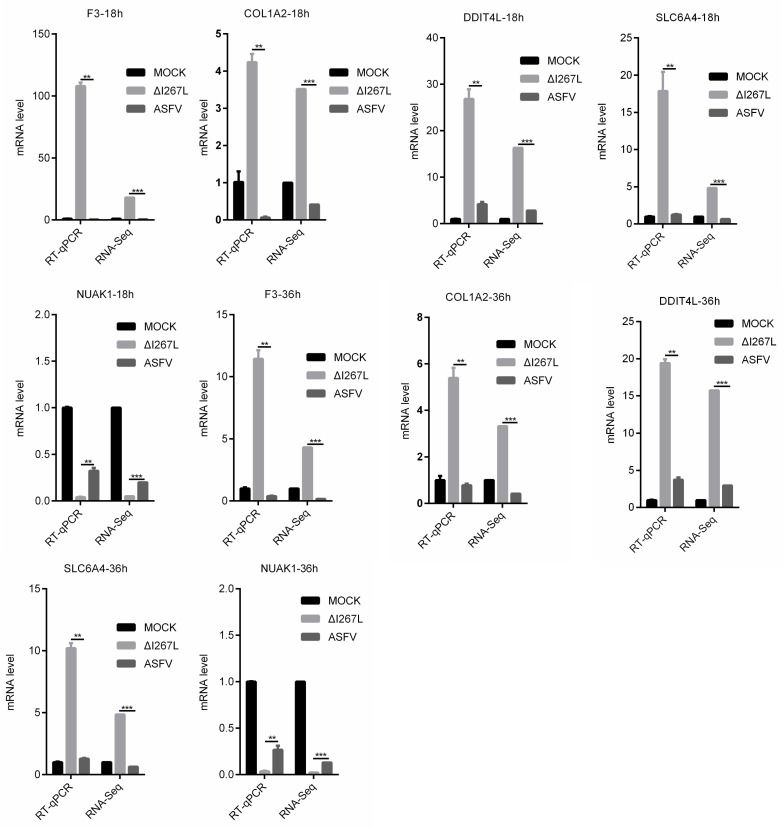
Validation of RNA-seq results through RT-qPCR. PAMs were uninfected or infected with ASFV or ∆I267L at a MOI of five; cells were harvested at 18 hpi. RNA was extracted and RT-qPCR was carried out to test their expression level. The results were calculated through the 2^−ΔΔCt^ method using porcine *GAPDH* as a reference. n = 3. ** *p* < 0.05, *** q < 0.05.

To validate the RNA-seq results, a RT-qPCR (PAMs and ASFV- or ∆I267L-infected PAMs) was used to examine the expression of the four most up-regulated DEGs (F3, COL1A2, SLC6A4, and DDIT4L) and the most down-regulated DEG (NUAK1) appeared at both 18 hpi and 36 hpi (Figure 6). The RT-qPCR results were largely consistent with the RNA-seq results, suggesting that the RNA-seq results were reliable.

### 3.7. I267L Can Inhibit TNF-α-Induced F3 Transcription

ASFV is a kind of virus that can greatly suppress the immune reaction, including the production of IL-1β and TNF-α production [16,47]. Based on our RNA analysis results, the transcription level of the major inflammation cytokines, such as TNF-α, IL-6, and IL-1β, were not significantly changed in ASFV- and ASFV–∆I267L-infected PAMs compared with uninfected PAMs (Supplementary Table S2), which indicated that infections in petri dishes had not induced the PAMs to secrete these cytokines, which was not the same result as infections in vivo.

Based on the RNA-Seq results and RT-qPCR results, we identified that the deletion of I267L affected the transcription of three coagulation-related genes, including F3, COL1A1, and COL1A2. As previous research found in infected pigs, inflammation cytokines such as TNF-α were greatly induced and could stimulate PAMs [48]; however, the way I267L functions under these conditions was still unknown. Therefore, we measured the transcription level of the three genes in ASFV/∆I267L-infected PAMs through TNF-α stimulation.

**Figure 7:**
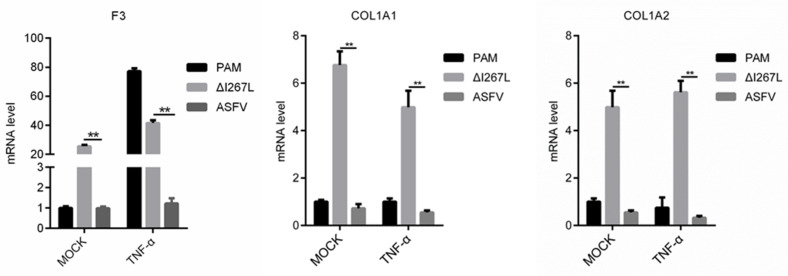
I267L can inhibit COL1A1/COL1A2/F3 transcription through TNF-α stimulation. PAMs were uninfected or infected with ASFV or ∆I267L at a MOI of five. PAMs were treated or untreated with TNF-α (10 pg/μL) at 6 hpi. At 18 hpi, cells were harvested to test their mRNA expression level through RT-qPCR. The results were calculated through the 2^−ΔΔCt^ method using porcine *GAPDH* as a reference. n = 3. ** *p* < 0.05.

Results (Figure 7) showed that the F3 transcription level was up-regulated in uninfected PAMs after stimulation using TNF-α. It was demonstrated that F3 was regulated by TNF-α. However, ASFV infection could inhibit TNF-α-induced F3 transcription, and the deletion of I267L partly impaired this inhibition. This implies that I267L has the ability to inhibit TNF-α-induced F3 transcription. For COL1A1 and COL1A2, I267L can also suppress their expression no matter whether TNF-α was to exist or not. These data indicate that I267L probably can also inhibit these three coagulation-related genes in vivo.

## 4. Discussion

As ASFV is continually transmitted across the Eurasian continent, more and more research is focusing on the virus. A series of pathologic genes have been identified; the I267L gene has been studied before. However, whether this gene belongs to pathogenic genes is still controversial. To elucidate the function of I267L, we firstly measured the growth kinetics of ∆I267L and found that it had a similar growth kinetic to the wild-type ASFV, indicating that I267L does not affect ASFV replication.

Transcriptome analysis is an efficient method for studying cell status. Using the gene-deleted virus in infected cells and comparing their transcriptomes with those of the wild type-virus-infected cells could provide us with clues about the gene’s function. In this study, we compared the transcriptome of ∆I267L-infected PAMs with ASFV-infected PAMs and identified 30 DEGs at 18 hpi and 38 DEGs at 36 hpi. Of these genes, 16 DEGs appeared at both time points, and F3, whose product is tissue factor, had the largest foldchange value. The deletion of I267L could significantly up-regulate the transcription level of F3, indicating that I267L could inhibit F3 expression and thus prevent the initiation of coagulation cascades, which is beneficial for the host to survive and produce more ASFV particles. However, the mechanism of this process is still unclear and needs further study. In addition, as the FVIIa:TF complex can induce pro-inflammatory effects in macrophages, the inhibition of F3 transcription can also alleviate inflammation in infected pigs.

Other hemorrhage-related viruses, such as classical swine fever virus [49], Ebola virus [50], influenza virus [51], and COVID-19 [52], can also lead to cytokine storms and increases in the expression of tissue factors. However, no virus gene has been found that modulates F3 directly. Our results showed that after infection, one ASFV gene could inhibit TF expression directly, thereby expanding our knowledge about the interaction between the virus and the host. Similar studies can also be applied to other hemorrhage-related viruses.

Apart from the coagulation cascades, the clotting process is always related to collagen-induced platelet activation [53]. Type I collagen is among the most abundant proteins in humans [54]; it is composed of two chains of collagen type I alpha 1 (COL1A1) and one chain of collagen type I alpha 2 (COL1A2) [55]. Therefore, COL1A1 and COL1A2 were also involved in blood coagulation. Based on the the RNA-Seq results, COL1A1 and COL1A2 were also significantly affected by the deletion of I267L. These data indicate that, separately from TF, I267L may inhibit the clotting process by blocking platelet activation. However, the mechanism for this physiological phenomenon needs further study.

Our data support how I267L is a hemorrhage- and immune-related gene through RNA-Seq analysis. Deletion of this gene may alter pathogenesis and can be tried as an attenuated strain. These things considered, I267L can also be deleted alongside other virulent genes to obtain an effective attenuate strain together, which is equivalent to the other attempt that was undertaken in the study of [56].

As our results showed that I267L is a virulent-related gene, we will now inspect the previous two reports on I267L. In the article that concluded that I267L was not a virulent gene, the pigs were inoculated with 100 HAD_50_ ΔI267L and wt-ASFV [34], while the pigs in the other article were challenged with 10 HAD_50_ ΔI267L and survived [35]. One proper explanation for this is that after the deletion of I267L, the strain was attenuated to some extent and was not lethal in 10 HAD_50_ but could still kill pigs at a higher viral dose (100 HAD_50_). In fact, in the first article, although the authors concluded that I267L was not a virulent gene, they also obtained the result from pigs inoculated with 100 HAD_50_ SY18DI267L, which presented higher rates of delayed fevers, viremia, clinical symptoms, and death compared with pigs inoculated with the same dose of ASFV SY18. This also suggests that deletion of I267L may attenuate the virulence of ASFV.

## 5. Conclusions

In this study, the function of I267L was studied through comparing the RNA-seq results of ASFV-infected PAMs and ∆I267L-infected PAMs. The DEG with the largest foldchange value was F3, which is the most important link between inflammation and coagulation cascades, thereby suggesting that I267 may affect hemorrhages of ASFV. In vitro experiments also showed that I267L could suppress TNF-α-induced F3 expression. GO analysis and KEGG analysis showed that the deletion of I267L could affect the immune system and the complement and coagulation systems. However, further studies are needed to elucidate this mechanism. Additionally, the effect of I267L on the immune system should also be studied to evaluate the role of I267L in ASFV infection.

## Figures and Tables

**Table 1:** Key software and parameters in RNA-seq analysis.

Software	Version	Parameters	Function
fastp	0.20.1	length_required 50	Raw nucleotide readings used for quality control
RseQC	4.0.0	default	RNA quality control
fastqc	v0.11.9	default	Raw readings used for quality assessment
Hisat2	2.1.0	--rna-strandness rf --fr	Genome comparison
samtools	1.9	mpileup –uRf –d 1,000,000	Analysis of sam file and bam file
HTseq-count	0.11.2	-s reverse	Gene quantification
DEGSeq	1.34.1	q value < 0.05, |log2FoldChange| > 1	Nonbiological repetitions used for difference analysis
clusterProfiler	v3.10.1	*p* value < 0.05	GO analysis and KEGG analysis

**Table 2:** Primers and oligonucleotides used in this study.

GenBank Number	Primers	Sequences
AF141959.1	porcine *GAPDH-F:*	ACATGGCCTCCAAGGAGTAAGA
	porcine *GAPDH-R:*	GATCGAGTTGGGGCTGTGACT
FR682468.2	p30-F:	CTCCGATGAGGGCTCTTGCT
	p30-R:	AGACGGAATCCTCAGCATCTT
AY504424.1	porcine F3-F:	CTGGAGCCACAGGCACTAC
	porcine F3-R:	GTCACACTCCGTGTCTGTCG
AB237775.1	COL1A2-F:	CTGGTCTTGGCGGGAACTTT
	COL1A2-R:	AGGACCAGTCTGACCAGGTT
CM000819.5	DDIT4L-F:	GAACTCCCAGCAGCGCC
	DDIT4L-R:	TCGTTGAGGTTGGGTTCAGG
CM000823.5	SLC6A4-F:	AGAATGAATGAGCTCGCCACC
	SLC6A4-R:	CGAATGGACGCTGACACACA
CM000816.5	NUAK1-F:	CCAGATGTGCAGTCCCCG
	NUAK1-R:	GCAGCATTGAGGAAGCAGC
CM000823.5	COL1A1-F:	AGCCCTGGTGAAAATGGAGC
	COL1A1-R:	AGCCCTGGTGAAAATGGAGC

**Table 3:** Statistics of the sequencing data quality.

Sample	Total Reads (M)	Clean Reads (M)	ASFV Mapped Percent	Swine Mapped Percent	Gene Number
PAM18 hpi	50.58	49.8	0.02	91.19	14240
ASFV18 hpi	47.85	47.02	9.61	78.97	14194
ΔI267L-18 hpi	50.17	49.27	9.92	78.08	14346
PAM-36 hpi	47.9	47.2	0.02	91.03	14177
ASFV36 hpi	49.49	48.36	25.10	48.05	13724
ΔI267L-36 hpi	48.94	48.09	27.47	52.90	13888

## Data Availability

All datasets generated for this study are included in the article.

## Supplementary Materials

The following supporting information can be downloaded at: https://www.mdpi.com/article/10.3390/microorganisms12020400/s1; Table S1. Fold changes of DEGs appeared at both 18 hpi and 36 hpi (∆I267L- vs. ASFV-infected PAMs); Table S2. Fold change values of three major inflammation cytokines; Figure S1. GO analysis at level 2 of the DEGs. (A) ΔI267L-infected PAMs vs. ASFV-infected PAMs at 18 hpi. (B) ΔI267L-infected PAMs vs. ASFV-infected PAMs at 36 hpi.; Figure S2. KEGG analysis for the distribution of the DEGs. (A) Distribution of DEGs for ΔI267L-infected PAMs vs. ASFV-infected PAMs at 18 hpi. (B) Distribution of DEGs for ΔI267L-infected PAMs vs. ASFV-infected PAMs at 36 hpi.
