# Supplementary material for: African Swine Fever Virus I267L Is a Hemorrhage-Related Gene Based on Transcriptome Analysis
Source: Microorganisms. 2024 Feb 17;12(2):400. doi: 10.3390/microorganisms12020400 (PMC10892147; doi:10.3390/microorganisms12020400)
Supplement: Supplementary file 1 [file microorganisms-12-00400-s001.zip › microorganisms-2773223-supplementary.pdf]

Supplementary materials

African Swine Fever Virus I267L Is a Hemorrhage-Related Gene Based on Transcriptome Analysis

Yuan Wen, Xianghan Duan, Jingjing Ren, Jing Zhang, Guiquan Guan, Yi Ru, Dan Li and Haixue Zheng

Table S1. Fold Changes of DEGs appeared at both 18 hpi and 36 hpi (ΔI267L- vs. ASFV-infected PAMs).

(ΔI267L- vs. ASFV- infected PAMs)		
**Gene name**	**18hpi**	**36hpi**
F3	40	27
COL1A2	9.3	8
SLC6A4	7.2	7.6
DDIT4L	6.4	5.3
AMCF-II	2.1	7
SLCO3A1	3.8	3.6
CISH	3.3	4
IL22RA2	3	4.2
IL1R1	2.3	4.6
LOC296866	3.6	3
B3GNT5	2.	3.3
LOC100153783	2.3	2.8
UPP1	2.3	2.5
BCL6	0.42	0.34
C3	0.36	0.29
NUAK1	0.42	0.17

Table S2. Fold Change values of three major inflammation cytokines.

Gene name	ASFV vs MOCK		ΔI267L vs MOCK	
	Foldchange value	Q value	Foldchange value	Q value
TNF (18h)	0.47	1	0.38	1
IL1B (18h)	4.64	1	2.24	1
IL-6 (18h)	Not identified	NA	776	1
TNF (36h)	0.97	1	1.34	1
IL1B (36h)	3.9	1	11	0.016
IL-6 (36h)	Not identified	NA	Not identified	NA

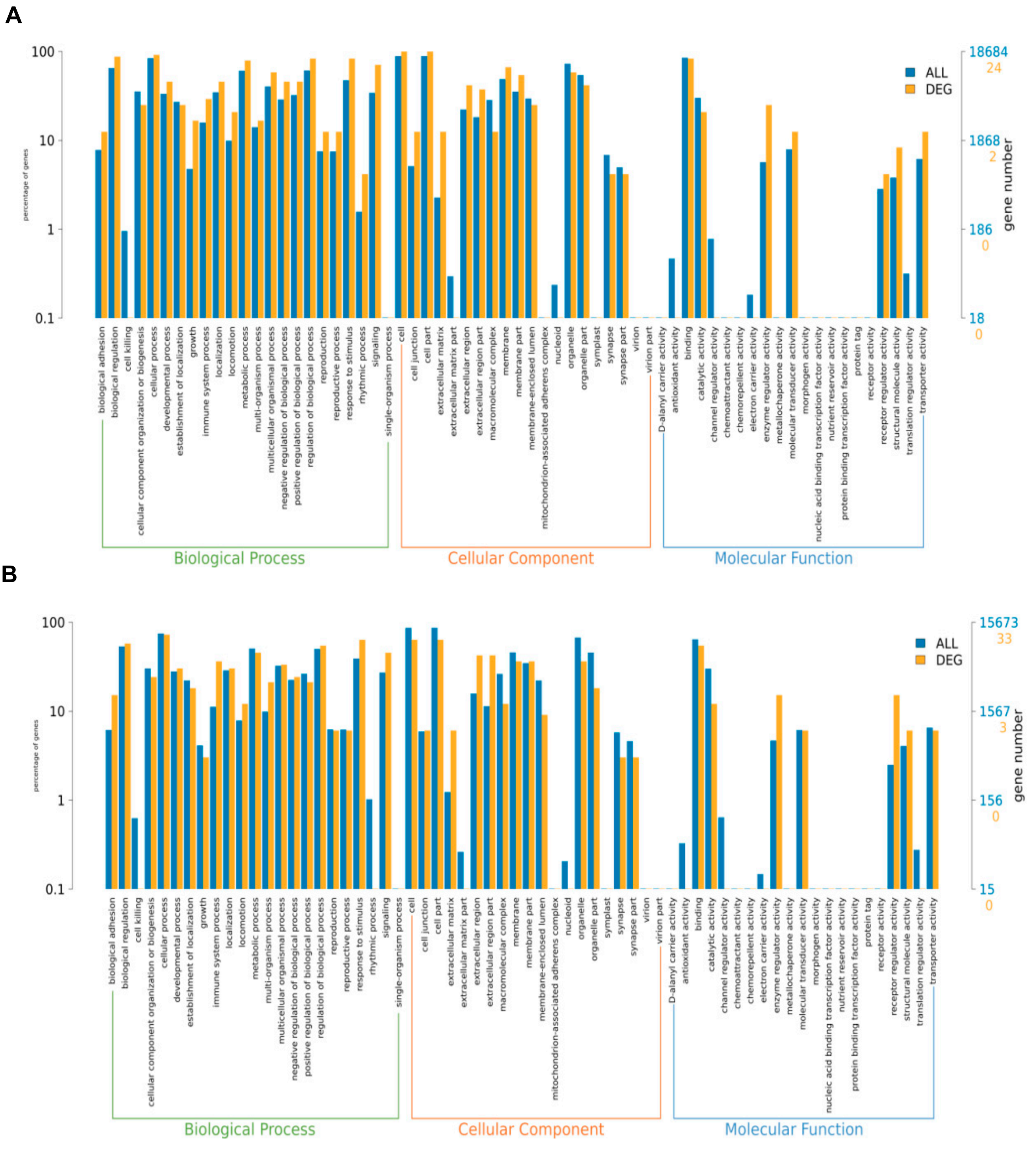

Figure S1. GO analysis at level 2 of the DEGs. (**A**) ΔI267L- infected PAMs vs. ASFV- infected PAMs at 18 hpi. (**B**) ΔI267L- infected PAMs vs. ASFV- infected PAMs at 36 hpi.

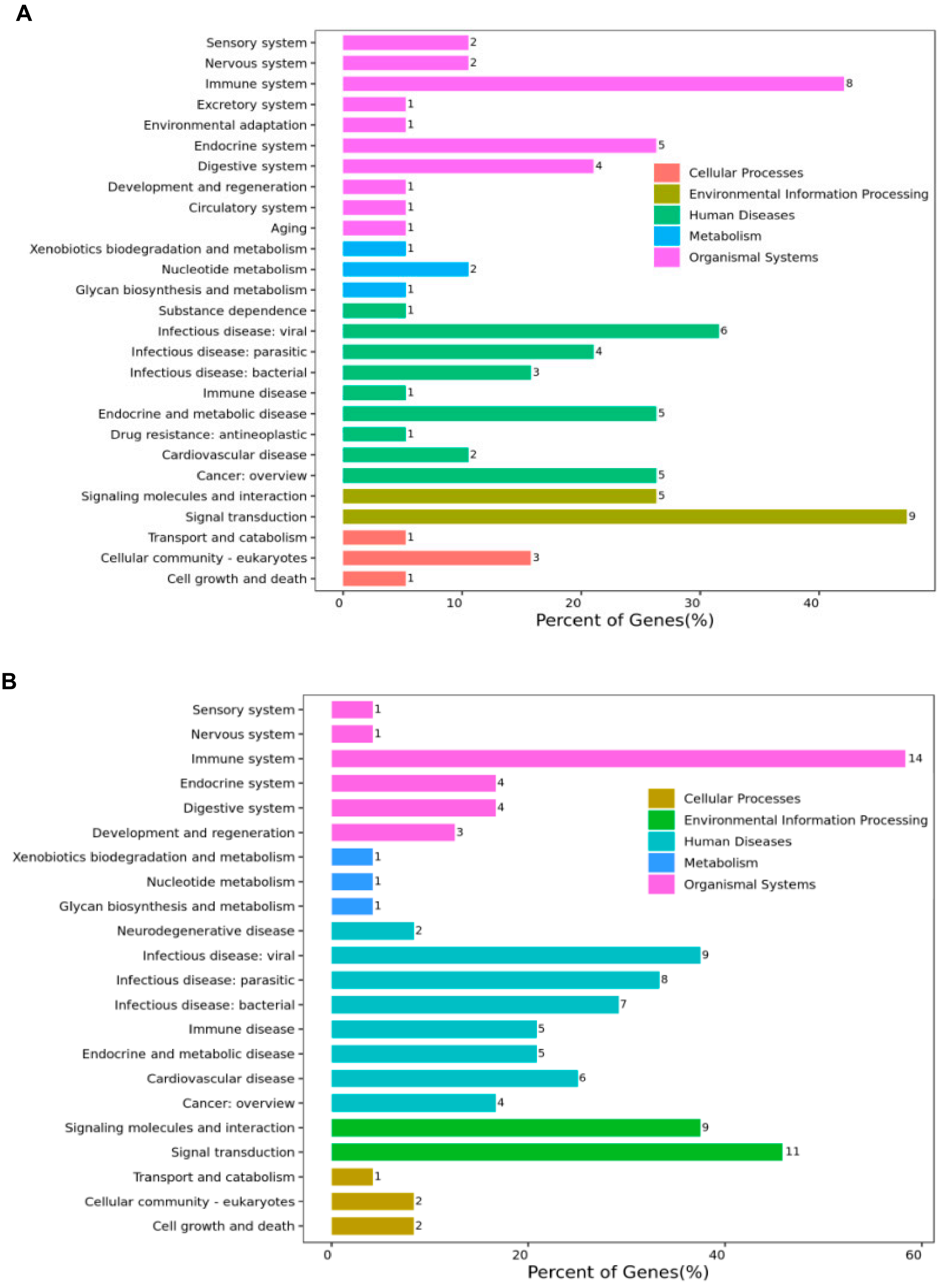

Figure S2. KEGG analysis for the distribution of the DEGs. (**A**) Distribution of DEGs for ΔI267L-infected PAMs vs. ASFV- infected PAMs at 18 hpi. (**B**) Distribution of DEGs for ΔI267L- infected PAMs vs. ASFV- infected PAMs at 36 hpi.
